# Complete mitochondrial genome of the crinoid echinoderm, *Florometra* species (Echinodermata, Crinoidea)

**DOI:** 10.1080/23802359.2020.1717390

**Published:** 2020-01-24

**Authors:** Sang-Eun Nam, Hyoung Sook Park, Jae-Sung Rhee

**Affiliations:** a Department of Marine Science, College of Natural Sciences, Incheon National University, Incheon, South Korea;; b Research Institute of Basic Sciences, Incheon National University, Incheon, South Korea

**Keywords:** Crinoid, echinoderm, Florometra, mitogenome

## Abstract

In this study, we report the sequence of the mitochondrial genome (mitogenome) of the crinoid echinoderm, *Florometra* species (Echinodermata, Crinoidea). The complete mitogenome of *Florometra* sp. was 15,792 base pairs long and was composed of 13 protein-coding genes (PCGs), two ribosomal RNA (rRNA) genes, 22 transfer RNA (tRNA) genes, and three regions of unassigned sequence (UAS) including one putative control region. Unique nucleotide composition, a clear positive bias for T with an apparent loss of C in PCGs as observed in the Crinoidea mitogenomes, was also seen in the *Florometra* sp. mitogenome (45% T, 12% C). Phylogenetic analysis with the concatenated nucleotide sequences of entire PCGs of echinoderms confirmed that *Florometra* sp. is highly related to *F. serratissima* within the family Crinoidea.

Echinoderms have been highlighted for numerous ecological and experimental researches, as they play a crucial role in structuring and maintaining marine ecosystems, particularly for the benthic community structure through foraging and grazing activities (Harrold and Pearse 1987; Pawson 2007; Uthicke et al. 2009). The five living echinoderm classes comprise Asteroidea (sea stars), Crinoidea (feather stars), Echinoidea (sea urchins), Holothruoidea (sea cucumbers), and Ophiuroidea (brittle stars). The family Crinoidea is one of the oldest clades of living echinoderms and has a fossil record spanning nearly half a billion years (Hess et al. 1999). The geologic history of crinoids and phylogenomics application facilitate opportunities to discuss on their evolution and developmental biology (Ausich 1980; Foote 1999; Janies 2001; Telford et al. 2014; Wright et al. 2017), while molecular information on crinoids is still insufficient to complement robust taxonomic status. Thus, accumulation of mitogenome information on the family Crinoidea will be useful to understand genetic diversity and molecular evolution of echinoderms.

In this study, we assembled the complete mitogenome of *Florometra* sp. (Accession no. MN883538) by employing Illumina HiSeq platform (Illumina, San Diego, CA, USA). An individual sample was collected from benthic zone (≈31 m in depth) at Yellow Sea (37°18′06.4″N, 126°18′19.8″E) using a plankton net. The voucher specimen was deposited in the Research Institute of Basic Sciences of Incheon National University and registered with unique specimen ID (201904-Crinoid014). Total genomic DNA was isolated using DNeasy Blood and Tissue kit (Qiagen, Hilden, Germany), followed by sequencing library generation with TruSeq RNA Sample Preparation Kit according to the manufacturer’s instructions (Illumina) and paired-end sequencing on Illumina HiSeq platform (Illumina) at Phyzen (Seoul, South Korea). Adapter sequences, low quality reads (sequences with >50% bases with quality value ≤5), reads with >10% of unknown bases, and ambiguous bases were totally removed to obtain high quality reads (Phred score of >20). The CLC *de novo* assemble algorithm was employed for assembly with CLC Assembly Cell package (version 4.2.1). Additional PCR procedure and Sanger sequencing were conducted to validate the Illumina-retrieved nucleotide sequences of *COI*, *cytB*, and UAS I region. The complete *Florometra* sp. mitogenome was annotated by using the MITOS web-based software (Bernt et al. 2013) and annotation result was confirmed with NCBI-BLAST (http://blast.ncbi.nlm.nih.gov).

The complete mitochondrial genome of *Florometra* sp. was 15,792 bp in length and comprised the typical set of 13 PCGs, 22 tRNAs, two rRNAs, and three UASs including one putative control region. Of mitogenomes published in crinoids, the highest sequence similarity in PCGs (≈88%) was exhibited to its closest relative with a mitogenome, *F. serratissima* mitogenome (Scouras and Smith 2001). Crinoid-specific bias in the nucleotide composition such as high T and low C ratio was also observed in *Florometra* sp. mitogenome as consisted of 26.9% A, 44.9% T, 12% C, and 16.1% G (Scouras and Smith 2001, 2006). Entire gene order with the positions of tRNAs and three UASs is identical to those of *F. serratissima* (Scouras and Smith 2001). All 13 protein-coding genes of 17 echinoderms including *Florometra* sp. were included in a maximum likelihood phylogenetic analysis, GTR + G + I model with a bootstrap of 1000 replicates (Figure 1). As a result, *Florometra* sp. is nested within Crinoidea, and highly supported (98%) as the sister species of *F. serratissima*.

**Figure 1.**
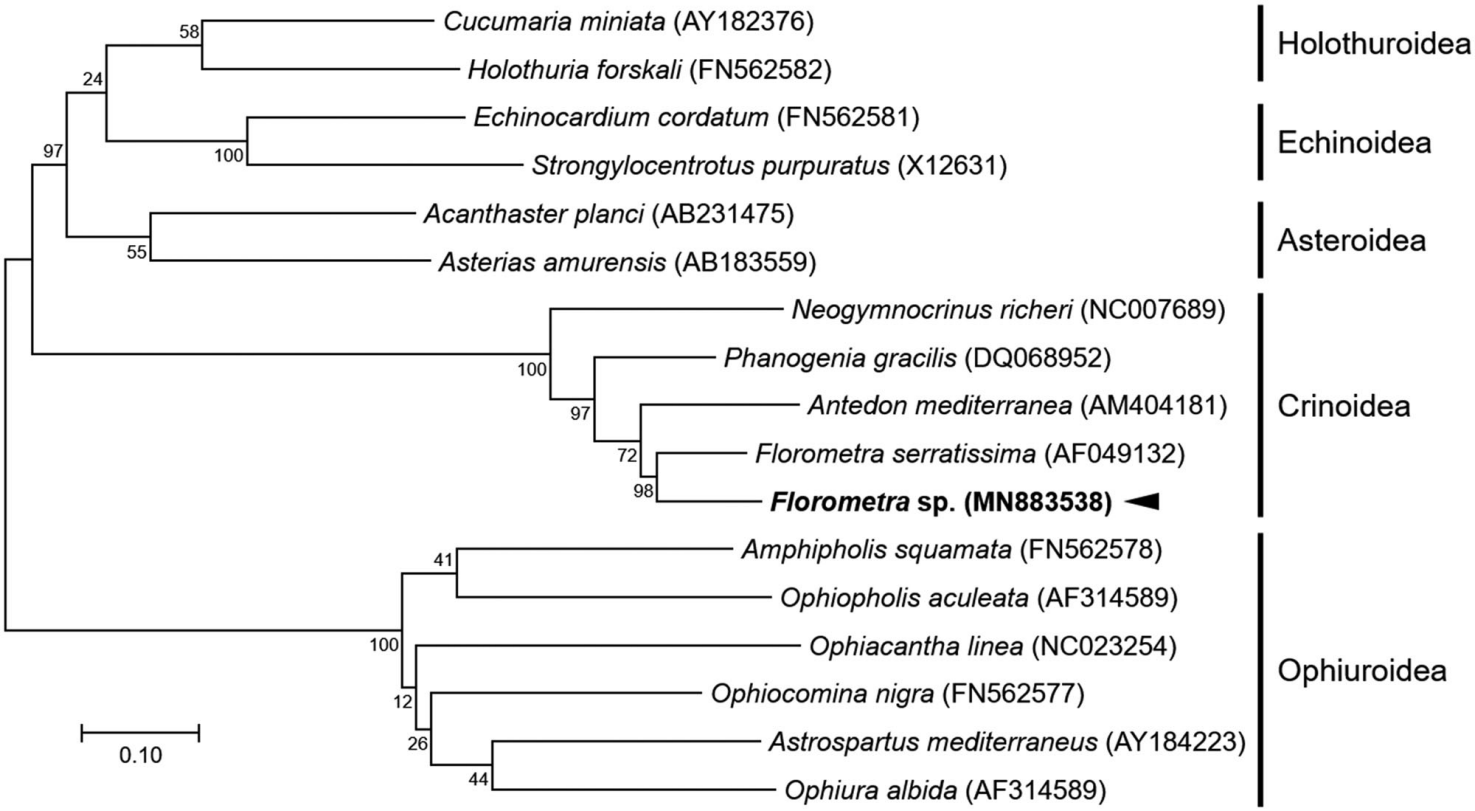
Maximum-likelihood (ML) phylogeny of 17 echinoderms (2 asteroids, 5 crinoids including *Florometra* sp., 2 echinoids, 2 holothuroids, and 6 ophiuroids) based on the concatenated nucleotide sequences of entire protein-coding genes (PCGs). Numbers on the branches indicate ML bootstrap percentages (1000 replicates). DDBJ/EMBL/Genbank accession numbers for published sequences are incorporated. The black arrow means the *Florometra* sp. analyzed in this study.
